# Supplementary material for: Two-dimensional gradients in magnetic properties created with direct-write laser annealing
Source: Nat Commun. 2025 Dec 9;16:10979. doi: 10.1038/s41467-025-65921-7 (PMC12689795; doi:10.1038/s41467-025-65921-7)
Supplement: Supplementary file 1 — Supplementary Information [file 41467_2025_65921_MOESM1_ESM.pdf]

Supplementary Information

Two-dimensional gradients in magnetic properties created with direct-write laser annealing

Lauren J. Riddiford, Jeffrey A. Brock, Katarzyna Murawska, Jacob Wisser, Xiaochun Huang, Nick A. Shepelin, Hans T. Nembach, Aleš Hrabec, Laura J. Heyderman

Contents

S1 Modeling laser-induced heating of thin films **2**

S2 Clarifying the competing physical transformations in materials systems **3**
S2.1 CoFeB Ferromagnets 3
S2.2 CoGd Ferrimagnets 6
S2.3 CoFeB/Pt/Ru SAFs 6
S2.4 Co/X/Co (X = Cr, Ta) SAFs 9

S3 Further magnetic characterization of films **11**

S4 Spin wave propagation in CoFeB **14**
S4.1 Details of micromagnetic simulations 14
S4.2 Comparison of simulation and experiment for different k-vectors 15
S4.3 Tunability of spin wave propagation bands 17

S5 Unprocessed images of domain wall motion in CoFeB/Pt/Ru SAFs **18**

S6 Further characterization of DWLA **19**

S1 Modeling laser-induced heating of thin films

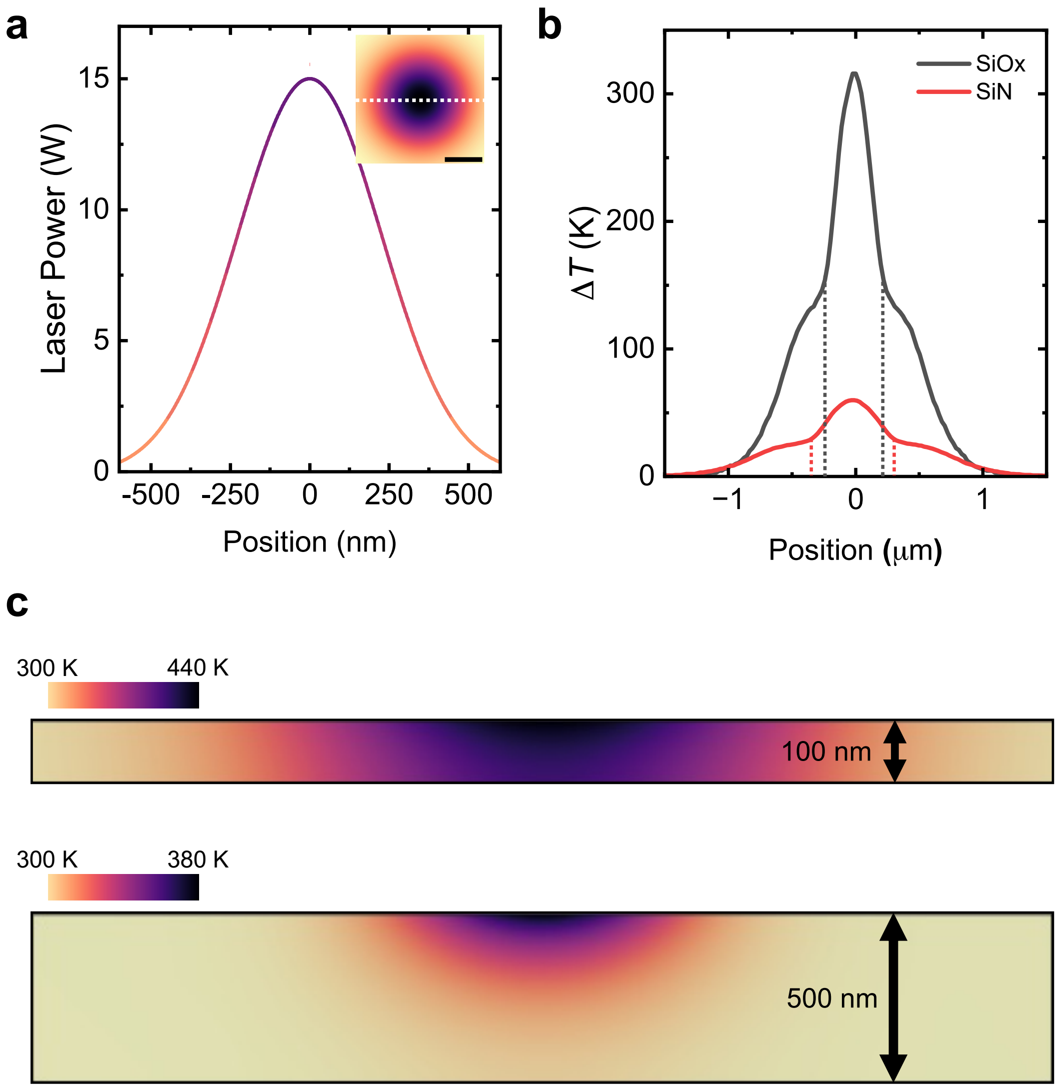

Supplementary Figure S1: Modeling laser-induced heating of metallic thin films. a, The spatial distribution of laser power input used to simulate heating resulting from DWLA. The inset shows a top-down image of the power profile. The plot shows the power profile along one direction, indicated by the white dashed line in the inset. The laser spot is assumed to have a Gaussian intensity profile, which is typical for a diode laser. Scale bar = 250 nm. **b,** The spatial variation in surface temperature after 10 ns of exposure for a 10 nm-thick Ta film on SiOx and on SiN using the laser power profile shown in a. The dashed lines denote the full width at half maximum of the two temperature profiles. The temperature change, ΔT, is given relative to the initial temperature of the sample (300 K), and the zero position corresponds to the center of the power profile shown in a. **c,** Cross-sectional views of the temperature profile for 100 nm and 500 nm-thick Ta films on SiOx substrates (substrates not shown) in response to the laser power input defined in a. These cross-sectional views are at the center of the heating profile shown in the inset of a, indicated by the white dashed line.

S2 Clarifying the competing physical transformations in materials systems

While we have highlighted the dominant physical transformation mechanism due to laser annealing in the main text, it is important to consider that other transformations may be taking place at the same time.

S2.1 CoFeB Ferromagnets

We argue the primary physical transformation when CoFeB/MgO is locally heated is the crystallization of CoFeB/MgO. To assess the possibility of interdiffusion, we perform x-ray reflectivity of as-grown films, a film annealed at a fluence that gives PMA (1.6 J cm^{-2}), and a film annealed at a fluence that is higher than the fluence that gives PMA (2.5 J cm^{-2}). For all fluences, the XRR data can be successfully modeled assuming that the interfacial and surface roughness remain unchanged compared to the as-grown material irrespective of the fluence. The data and corresponding fits generated with GenX are shown in Supplementary Fig. S2. This rules out any significant increases in interfacial roughness or interdiffusion as a result of laser annealing.

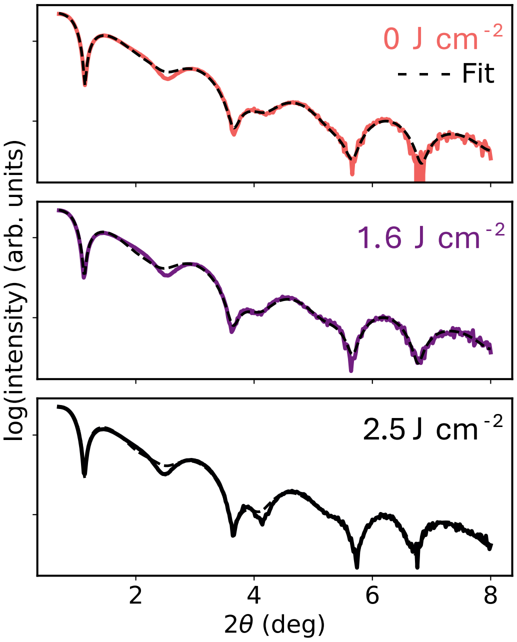

Supplementary Figure S2: X-ray reflectivity data of CoFeB films. X-ray reflectivity data for a single film as-grown (upper panel), annealed at 1.6 J cm^{-2} (middle panel), and annealed at 2.5 J cm^{-2} (lower panel). While the density of the layers changes slightly, the interfacial and surface roughness of the films do not change significantly enough to alter the quality of the x-ray reflectivity fit (see Methods Section 3.4). This means that there is not significant interdiffusion in this system as a result of laser annealing.

We examine possible oxidation of the CoFeB layer during laser annealing with SQUID-VSM measurements. In Supplementary Fig. S3, we observe that the saturation magnetization does not change when going from the as-grown film to the film annealed at 1.6 J cm^{-2}, the fluence which gives perpendicular magnetic anisotropy. At a higher fluence of 2.5 J cm^{-2}, the saturation magnetization has decreased, suggesting oxidation that reduces the magnetic moment of the CoFeB. Note that the fluences here differ from those in Fig. 3b in the main text. This is because this film was grown on a different SiOx wafer that had a different thermal conductivity, leading to different optimal fluences for structural and magnetic transformation.

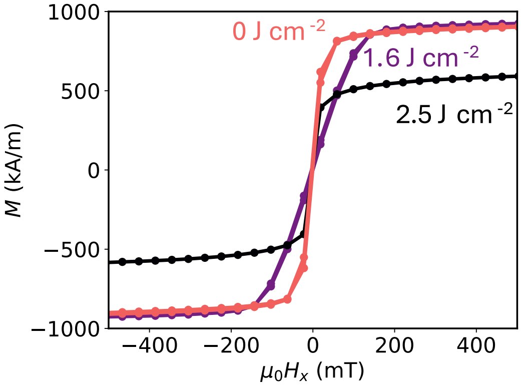

Supplementary Figure S3: Magnetization of CoFeB films. Magnetization vs applied in-plane magnetic field for a CoFeB film, as-grown and annealed at two different fluences, measured by SQUID-VSM. The uncertainties in the magnetization measurements are smaller than the symbols used (see Section 3.5 of the Methods on magnetic characterization).

Finally, cross-sectional transmission electron microscope (TEM) images of larger regions of the CoFeB lamellae used for TEM images in Fig. 1a of the main text are shown in Supplementary Fig. S4, indicating that the CoFeB/MgO film has crystallized.

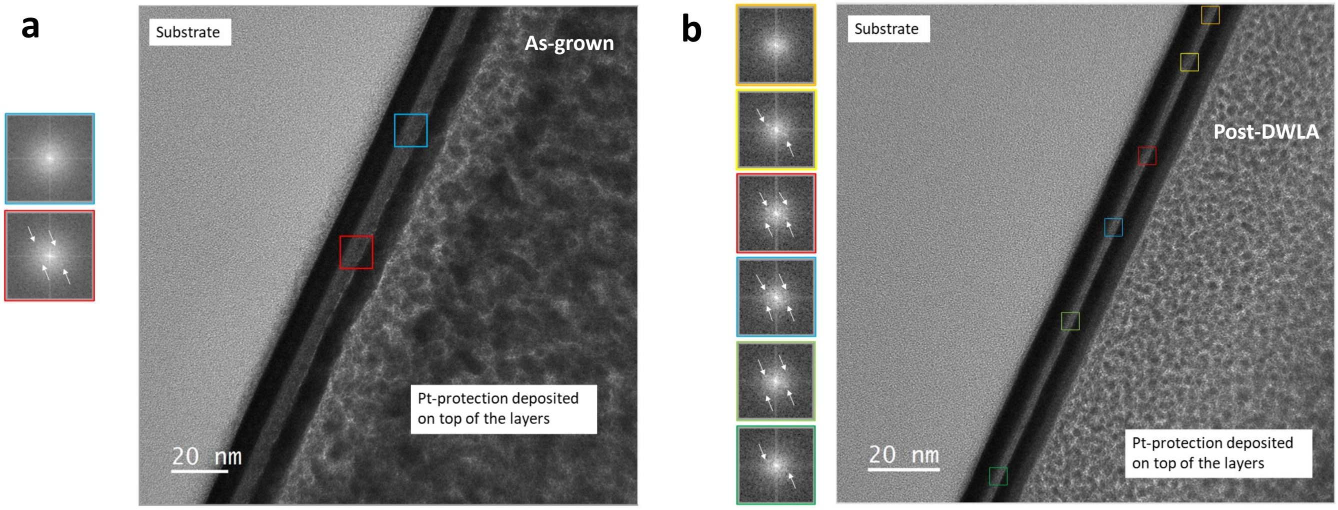

Supplementary Figure S4: TEM of CoFeB before and after DWLA. Extended cross-sectional TEM images of **a,** as-grown and **b,** CoFeB thin films laser annealed at a fluence of 2.5 J cm^{-2}. Electron diffraction patterns are shown for different points along the lamella. We searched for diffraction peaks across each lamella. **a,** For the as-grown film, we find one location where there are very weak diffraction peaks (indicated by the white arrows in the red box), but everywhere else, no peaks are evident (with the blue box as a representative example). **b,** After laser exposure, weak diffraction spots are visible across most of the cross-section of the film (indicated by arrows in the boxes of selected regions along the cross section). Exceptions are the top region (orange box), which does not show clear diffraction spots, and the bottom region (green box) which has particularly weak, diffuse spots. While the strength of the diffraction spots varies depending on the region selected, their appearance indicates that the CoFeB/MgO film has crystallized.

S2.2 CoGd Ferrimagnets

In the case of the CoGd ferrimagnets, a change in the crystal structure of the heterostructure may accompany the change in oxidation detected in response to DWLA, which could also modify the magnetic properties. To probe any changes in the crystallinity, we performed XRD measurements of a CoGd film before and after DWLA treatment using a laser fluence of 1.6 J cm^{-2}. As is typical for amorphous CoGd ferrimagnets, the as-grown film exhibits a broad peak corresponding to the (111) polycrystalline texture of the Pt seed layer and an intense, narrow peak from the Si (400) reflection of the substrate (blue curves in Supplementary Fig. S5a,b). After uniform DWLA treatment, there are no obvious changes with no new peaks appearing (red curves in Supplementary Fig. S5a,b), indicating that the CoGd layer remains macroscopically amorphous. Furthermore, the width of the rocking curve collected about the Pt(111) reflection does not show a strong change after DWLA treatment (compare blue and red curves in Supplementary Fig. S5c), signifying that the polycrystalline texture of the Pt seed layer is not affected by DWLA.

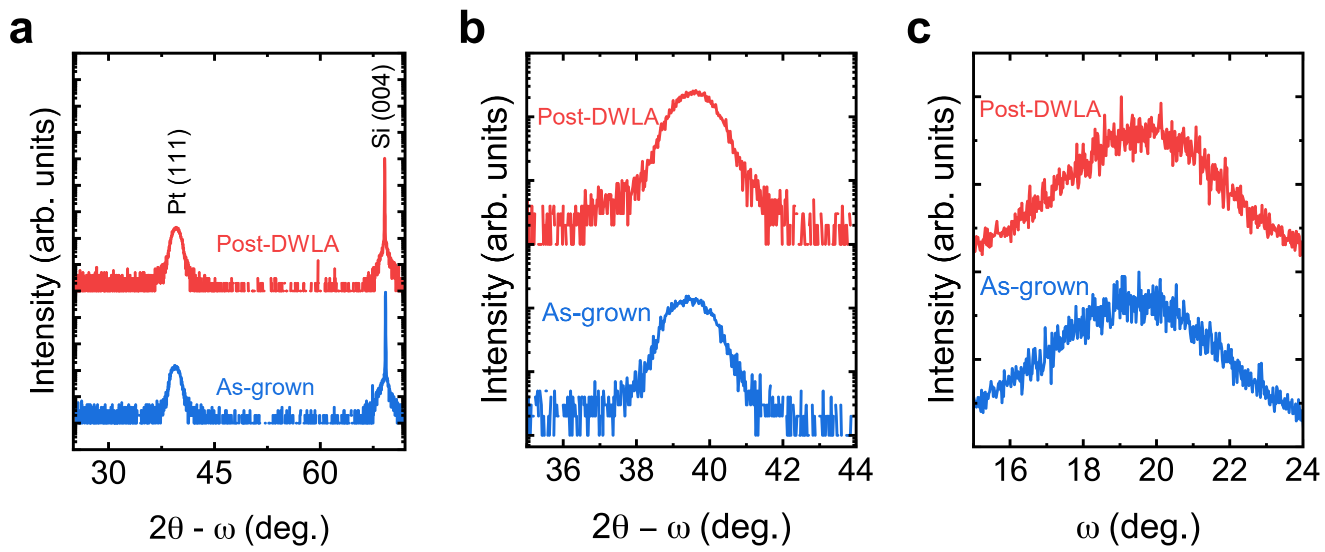

Supplementary Figure S5: X-ray characterization of CoGd. a,b XRD spectra of a CoGd film in the as-grown state and after uniform DWLA treatment using a laser fluence of 1.6 J cm^{-2} with the x-ray spectra taken over a large angular range (a) and in the vicinity of the Pt (111) peak (b). The ω offset was set by maximizing the intensity of rocking curves collected about the Si (400) reflection. **c,** Rocking curves measured about the Pt (111) reflections shown in a,b.

S2.3 CoFeB/Pt/Ru SAFs

For CoFeB/Pt/Ru SAFs, we have identified interdiffusion as the dominant transformation mechanism. From XRD measurements, it is clear that the Pt crystallinity changes due to this interdiffusion, so these two mechanisms are linked, but the degree of crystallinity in the system does not seem to change significantly. In particular, the x-ray diffraction peaks shown in Fig. 2c of the main text are the only film peaks detected over a broad range of 2θ values for both the as-grown film and the film subjected to DWLA at a fluence of 3.9 J cm^{-2}, as seen in Supplementary Fig. S6.

We eliminate significant oxidation of the magnetic layers by comparing the film saturation magnetization before and after annealing (seen in Supplementary Fig. S7). We find that, after annealing at a fluence of 2.9 J cm^{-2}, there is a 10% decrease, which is likely to be the result of interdiffusion

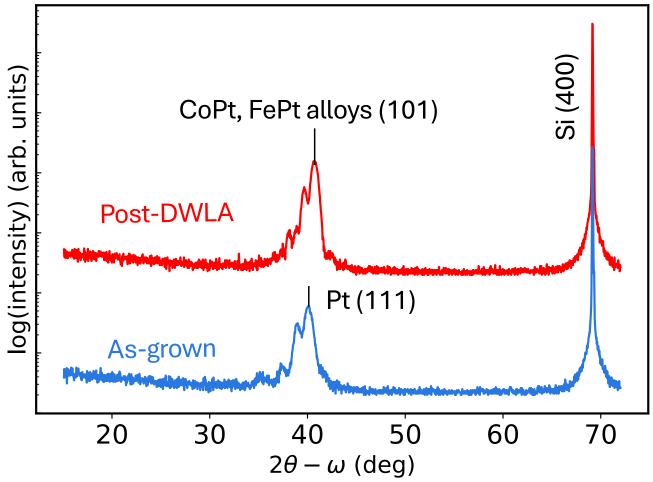

Supplementary Figure S6: X-ray diffraction $2\theta - \omega$ scans of CoFeB/Pt/Ru SAFs. The $2\theta - \omega$ scans over a large angular range of a CoFeB/Pt/Ru SAF are shown for the as-grown film (in blue) and the film annealed at a fluence of 3.9 J cm^{-2} (in red).

of CoFeB and Pt. Regarding oxidation, it would be difficult for oxygen to penetrate deep into this heterostructure due to the many Pt layers, which form robust barriers to oxidation. If there was some oxidation taking place, we would expect it to affect the upper magnetic layer more than the lower magnetic layer. A smaller saturation magnetization in the top magnetic layer would lead to an increased remanent net magnetization of the heterostructure because of the antiferromagnetic alignment of the magnetization in the two layers. However, the remanent magnetization after annealing decreases from 260 kA/m to 235 kA/m (see inset in Supplementary Fig. S7a), indicating that the saturation magnetization of the upper magnetic layer has not decreased any more than the saturation magnetization of the lower layer. Thus, oxidation is likely to be minimal in this heterostructure.

We also use remanent magnetization measurements to verify that the observed change in the anisotropy of the SAF is due to a change in anisotropy in both magnetic layers. From the central region of the hysteresis loop in the inset of Supplementary Fig. S7a, it can be seen that the remanent magnetization of the laser annealed film is also 10% lower than that of the as-grown film. To explain this reduction in the remanent magnetization, one can think of two possible scenarios for the magnetic configuration after annealing, given by the schematics to the right which depict the magnetization in the upper and lower magnetic layers before and after laser annealing. In the as-grown magnetic configuration (upper schematic), the two layers have antiparallel magnetization directions. Because the lower magnetic layer has a higher saturation magnetization, there is a low, non-zero net magnetization at zero field. In Scenario 1 (middle schematic), the PMA of the top magnetic layer is weakened, while the lower magnetic layer is unaffected. In this case, the remanent magnetization of the laser annealed film would increase because the magnetization of the lower layer is not offset by an opposing out-of-plane magnetization in the top layer. In Scenario 2 (lower schematic), laser annealing impacts the anisotropy of both magnetic layers, so that the anisotropy field associated with the PMA in each layer is reduced by the same amount. In this case, the remanent magnetization would be unaffected, as the magnetization of the two layers remains antiparallel. Because the remanent magnetization does not increase with laser annealing, we conclude that Scenario 2 is most likely.

From in-plane SQUID-VSM measurements of the same SAF films seen in Supplementary Fig. S7b,

we can extract the change in the effective anisotropy energy, which decreases from 5.4×10^5 J m^{-3} to 3.9×10^5 J m^{-3}. The fluences for these films measured in SQUID-VSM differ from those in Fig. 3d in the main text. This is because this film was grown on a different SiOx wafer that had a different thermal conductivity, leading to different optimal fluences for structural and magnetic transformation.

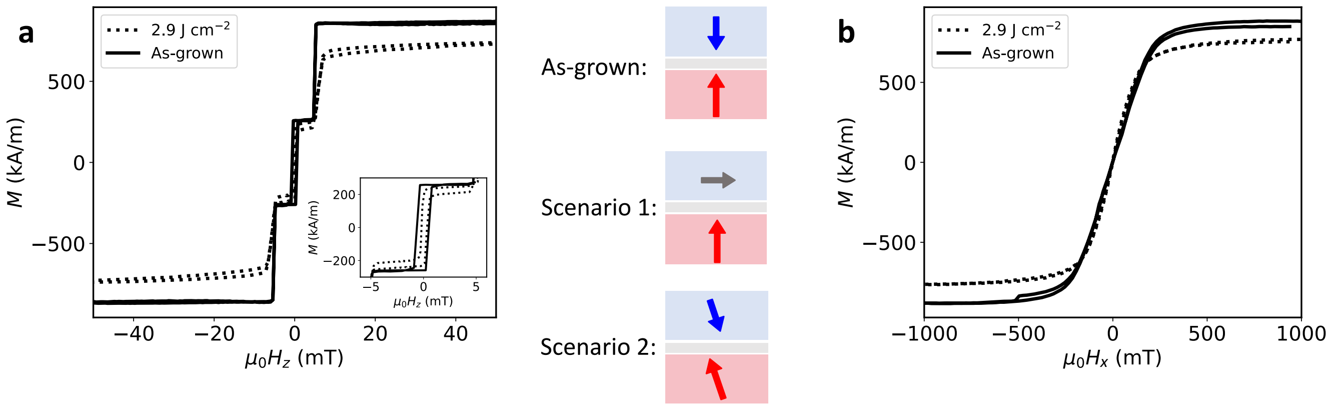

Supplementary Figure S7: Magnetometry measurements of CoFeB/Pt-SAF films. Room temperature SQUID-VSM measurements of two identical SAF films, one as-grown and one that has been laser annealed at a fluence of 2.9 J cm^{-2}. **a,** Out-of-plane magnetometry reveals that the saturation magnetization of the laser annealed sample is $\sim$ 10 % lower than that of the as-grown film. Inset: the remanent magnetization of the films, which is also $\sim$ 10% lower after laser annealing. The schematics to the right indicate (upper) the magnetic configuration before annealing and (middle and lower) the two possible magnetic configurations after annealing. **b,** In-plane magnetometry of the laser annealed and as-grown SAF films. The effective anisotropy field is lower for the annealed sample, and the saturation magnetization of the laser annealed sample is confirmed to be 10% lower than the as-grown film. The standard error of the measurements shown is smaller than the lines used (see Section 3.5 of the Methods on magnetic characterization).

S2.4 Co/X/Co (X = Cr, Ta) SAFs

Interdiffusion in the Co/X/Co (X = Cr, Ta) SAFs as a result of DWLA was confirmed using dynamic SIMS characterization. We note that the composition of the sample used for SIMS characterization was different to that stated in Section 3.1 of the Methods on synthesis, and has the composition Si/SiOx (300 nm)/ Co (10 nm)/ Cr (10 nm). By increasing the thickness of the Co and Cr layers above the values given in the main text, we increased the number of etching cycles over which a significant signal could be detected for each element during the measurement. Omitting the top Co layer reduces the number of Co/Cr interfaces being probed, thus simplifying the analysis of the results. Comparison of the as-grown and post-DWLA SIMS profiles (blue and red curves in Supplementary Fig. S8a, respectively) reveals that, after DWLA using a laser fluence of 2.5 J cm^{-2}, Co is detected after significantly fewer etching cycles, and signals from both Co and Cr persist over a broader range of etching cycles. In concert, these findings indicate that DWLA causes Co to interdiffuse into the Cr layer. It should be noted that oxidation at the surface of the Cr layer affects the initial sputter yield of Cr. The impact of Co-Cr interdiffusion on the magnetic properties is supported by measurements of the RKKY coupling field as a function of Co content in a series of films in which the Cr layer was deliberately alloyed with Co during the growth process by co-sputtering a Co-Cr spacer layer (Supplementary Fig. S8b). This shows that interdiffusion of Co into Cr can lead to ferromagnetic coupling between Co layers separated by Cr.

To assess whether DWLA alters the crystal structure of the RKKY spacer layer, x-ray diffraction measurements were performed on a Si/ SiOx (300 nm)/ Ta (10 nm) system before and after DWLA using a laser fluence of 2.5 J cm^{-2}. Here, the Ta layer was deposited by DC sputtering. The 2θ–ω scan of the as-grown sample, taken over a large angular range (blue curve in Supplementary Fig. S8c), exhibits two diffraction peaks: A diffuse peak at a low angle corresponding to the (002) reflection of β-Ta, and a sharp peak at a higher angle corresponding to the (400) reflection of the Si substrate. No additional diffraction peaks are observed following DWLA (red curve in Supplementary Fig. S8c), indicating that no new crystalline phases are formed. Furthermore, the width of rocking curves about the β-Ta (002) reflection remains unchanged after DWLA (Supplementary Fig. S8d), suggesting that DWLA does not affect the polycrystalline texture of Ta.

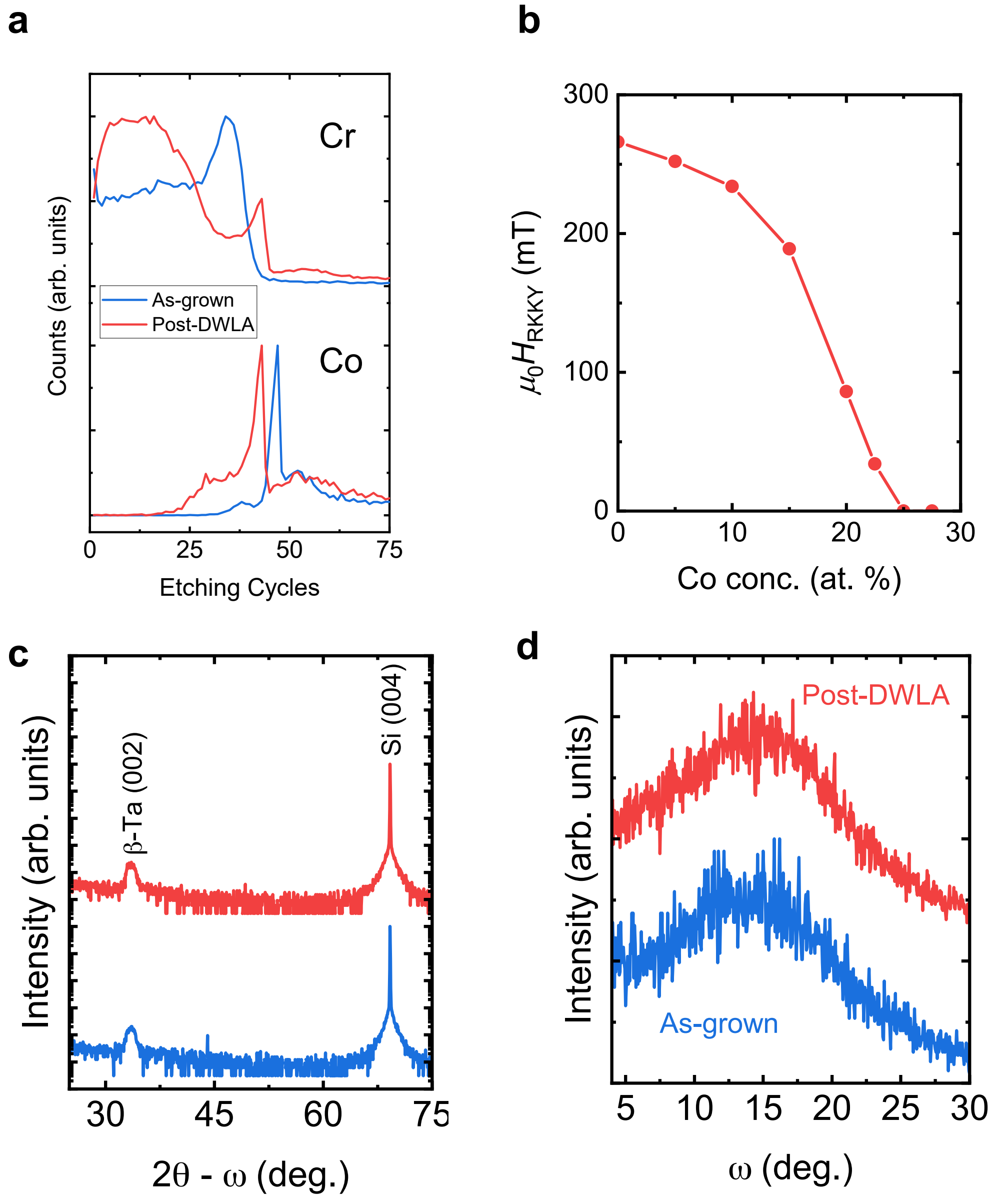

Supplementary Figure S8: Chemical and structural characterization of Co/X/Co (X = Cr, Ta) SAF films a, Cr- and Co-sensitive SIMS profiles for an as-grown Si/ SiOx (300 nm)/ Co (10 nm)/ Cr (10 nm) sample (blue curves) and after DWLA treatment using a laser fluence of 2.5 J cm^{-2}(red curves). **b,** Strength of antiferromagnetic RKKY exchange coupling field $\mu_0 H_{\mathrm{RKKY}}$ as a function of Co concentration x in a Co/Co_xCr_{1-x}/Co SAF (where x denotes an atomic percentage), determined using pMOKE magnetometry. The hysteresis loops from which $\mu_0 H_{\mathrm{RKKY}}$ was extracted are similar to those shown in Supplementary Fig. S9, and are provided in the Zenodo repository associated with this work. **c,** XRD spectra of a SiOx/ Ta (10 nm) film in the as-grown state and following uniform DWLA treatment using a laser fluence of 2.5 J cm^{-2}. The ω offset was chosen by maximizing the intensity of rocking curves collected about the Si (400) reflection. **d,** Rocking curves measured about the Ta (002) reflections visible in c.

S3 Further magnetic characterization of films

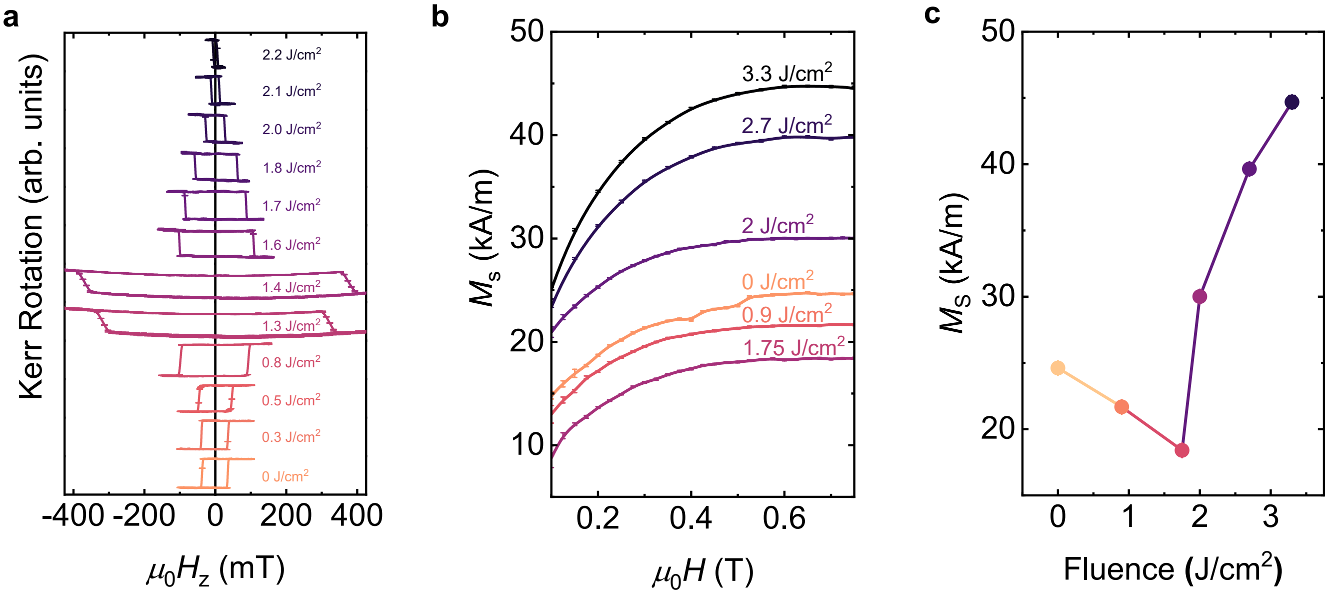

Supplementary Figure S9: Magnetic characterization of CoGd films. a, Room temperature pMOKE hysteresis loops for 100 µm × 100 µm regions of a CoGd film exposed with various laser fluences, collected using MOKE microscopy. The error bars in the Kerr Rotation at each data point were determined using the procedure detailed in Section 3.3 of the Methods on Magneto optic Kerr effect measurements. **b,** Room temperature, in-plane SQUID-VSM measurements of M vs $\mu_0 H$ for continuous CoGd films that were uniformly exposed using the indicated laser fluences. Details on how the error bars for the magnetization values were determined is provided in Section 3.5 of the Methods on Magnetic characterization. **c,** Saturation magnetization as a function of laser fluence extracted from b. The uncertainties in the saturation magnetization measurements are smaller than the symbols and lines used (see Section 3.5 of the Methods on magnetic characterization for information on how the error bars were determined).

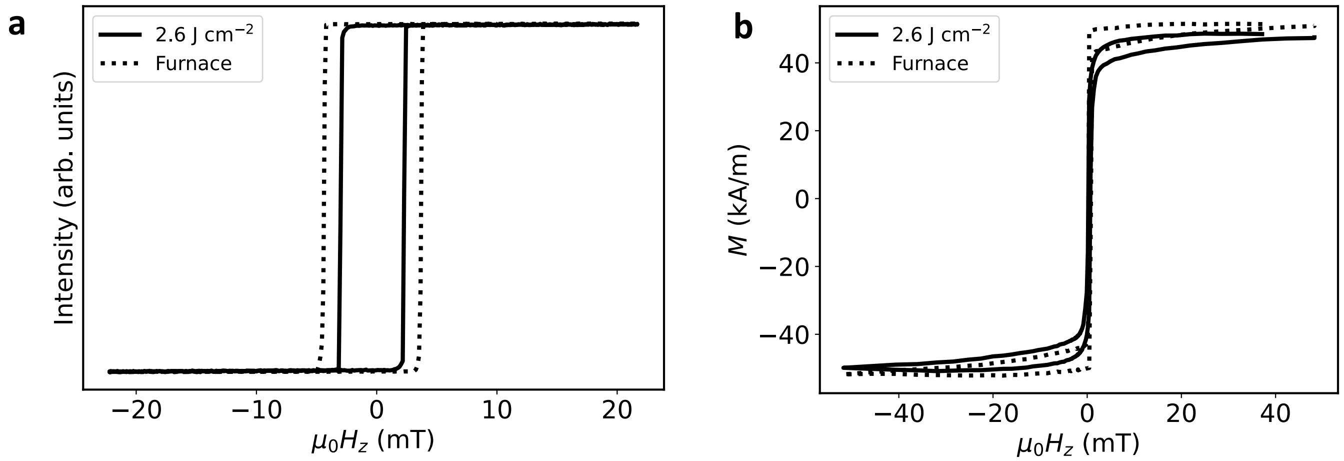

Supplementary Figure S10: Comparison of CoFeB magnetic properties subjected to laser annealing and furnace annealing. Identical CoFeB films were laser annealed and furnace annealed (in a tube furnace at atmosphere for 1 hour at 300 °C with a temperature ramp rate of 20 °C/minute). **a,** pMOKE microscopy measurements of the two samples reveals hysteresis loops with similar square shapes, with only a small difference in the coercive field. **b,** Out-of-plane SQUID-VSM measurements for both samples are also very similar. The saturation magnetization is the same for both samples, indicating that the two techniques give an equivalent transformation of magnetic properties in CoFeB. The standard errors of the measurements shown are smaller than the lines used (see Section 3.5 of the Methods on magnetic characterization).

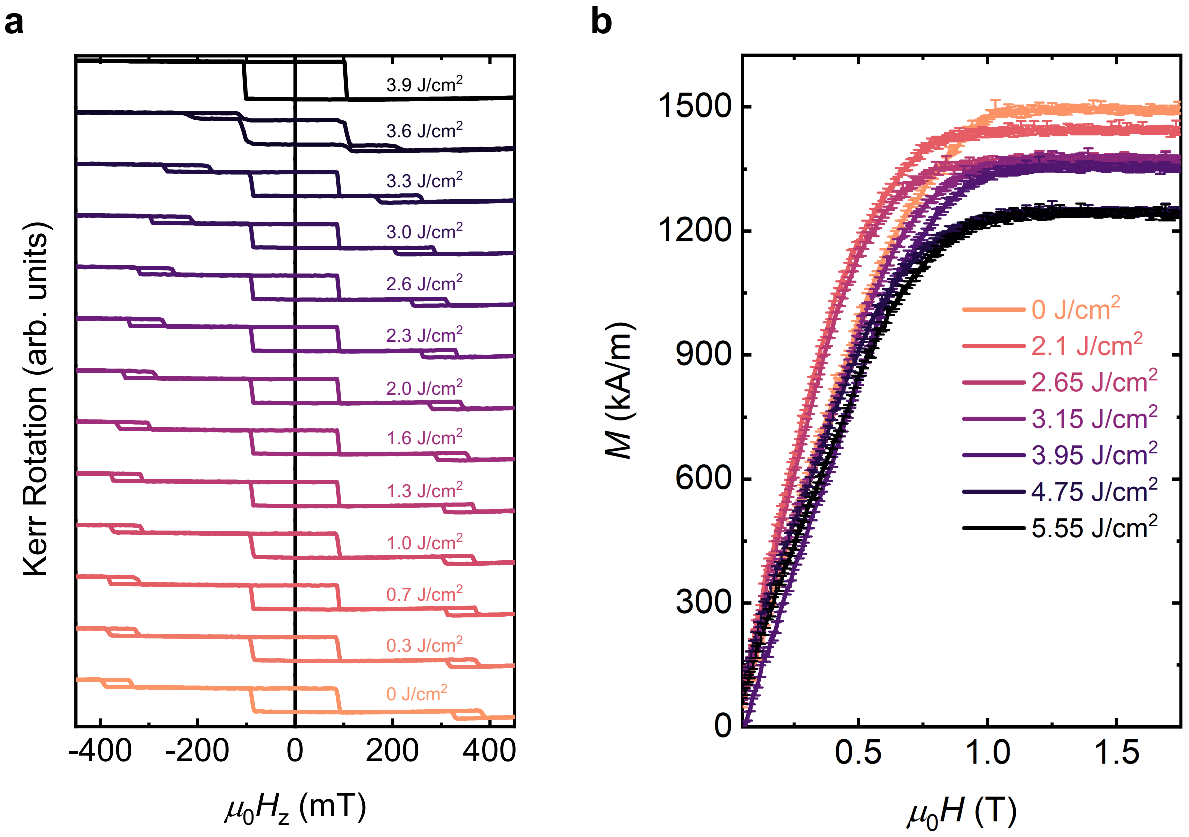

Supplementary Figure S11: Full magnetic characterization of Co/Cr/Co SAF films. **a,** Room temperature, pMOKE hysteresis loops of a Co/Cr/Co SAF sample obtained using MOKE microscopy along the spiral-shaped magnetic structure with a laser exposure gradient shown in the inset of Fig. 3c of the main text. The loop corresponding to each fluence was collected by selecting a 5 µm × 5 µm area at specific points along the spiral and only using light reflected from this area to obtain the hysteresis loop. Fluence values were assigned by mapping the location of each exposed area to the laser fluence design shown as a color overlay within the inset of Fig. 3c. The error bars for the Kerr Rotation at each data point were determined using the procedure detailed in Section 3.3 of the Methods on Magneto optic Kerr effect measurements. **b,** Room temperature, in-plane M vs $\mu_0 H$ measurements taken with SQUID-VSM of continuous Co/Cr/Co SAF films that were uniformly exposed using the indicated laser fluences. See the Section 3.5 of the Methods on magnetic characterization for details on how the error bars were determined.

S4 Spin wave propagation in CoFeB

S4.1 Details of micromagnetic simulations

Spin wave propagation in patterned CoFeB films was simulated using mumax3. In order to have a reasonable simulation time, we simulate an 18 µm × 3 µm × 1.3 nm volume (simulation volume and coordinate system shown in Supplementary Fig. S12), divided into cells with dimensions x × y × z = 5 nm × 5 nm × 1.3 nm. The simulation parameters are M_s = 975 kA/m, A_{ex} = 10 pJ m^{-1} [1], and $\alpha = 0.02$. We consider two geometries, representing spin waves traveling along a stripe (Supplementary Fig. S12a) and spin waves traveling orthogonal to six 3 µm-wide stripes (Supplementary Fig. S12b). For the stripe in Supplementary Fig. S12a, K_{u1} is varied linearly from 4.4×10^5 J m^{-3} to 6.2×10^5 J m^{-3} in steps of 3.6×10^3 J m^{-3} (from light blue to dark purple) across 50 regions with dimensions of 18 µm × 60 nm. The anisotropy gradient is the same for the stripes in Supplementary Fig. S12b, except that the region dimensions are 60 nm × 3 µm.

A magnetic field H (0.15 T, 0.263 T, or 0.35 T) is applied along the y direction (see Supplementary Fig. S12) and the configuration is relaxed in the presence of the field. The field is kept in place and a 2 mT sinc pulse over 20 ns is applied in a 10 nm-wide excitation region in the middle of the structure (indicated by the yellow box in Supplementary Fig. S12). The vector magnetic moment for each cell is saved every 2.5×10^{-11} s. To reduce reflections, three regions are defined at x = ±(7 µm − 9 µm), each with dimensions of 660 nm × 3 µm and damping of 0.05, 0.2, and 1 (light, mid, and dark gray boxes in Supplementary Fig. S12). An additional in-plane uniaxial anisotropy contribution of $K_{\mathrm{IP}} = 4.3 \times 10^4$ J m^{-3} was introduced along the gradient stripe using the custom fields function in mumax3 as detailed in Ref. [2].

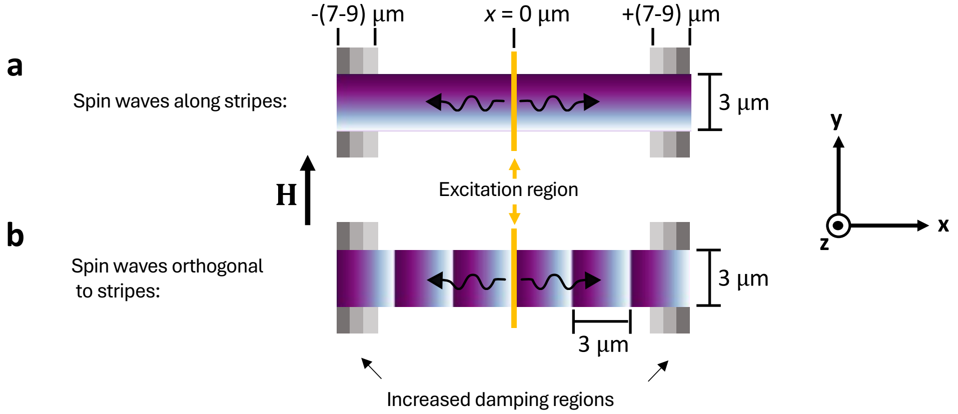

Supplementary Figure S12: Schematic of geometries used for micromagnetic simulations. Two geometries are simulated using mumax3. In **a**, the spin waves propagate along the stripes, while in **b**, the spin waves propagate orthogonal to the stripes. For both geometries, a 10 nm-wide excitation region is placed in the middle (in yellow, not shown to scale), regions at the edges have increased damping to decrease reflections, and a magnetic field H is applied along the y direction.

S4.2 Comparison of simulation and experiment for different k-vectors

Propagation of spin waves along stripes: To verify the agreement between simulated and measured spin wave propagation, the fast Fourier transform (FFT) intensity of the simulated system is compared to the BLS intensity measured at several different k values. We compare these values for a magnetic field of 0.35 T applied orthogonal to the stripes (for experimental and simulation geometries, see Fig. 4a and Supplementary Fig. S12a, respectively), where the BLS signal (9.8 GHz – 13.3 GHz) is separated from low-frequency phonon modes (near 0 GHz). We observe that the frequency range where BLS intensity is measured is almost identical to that simulated using mumax3. Qualitatively, we also see a higher intensity at high frequency compared to the other frequencies in both simulation and experiment.

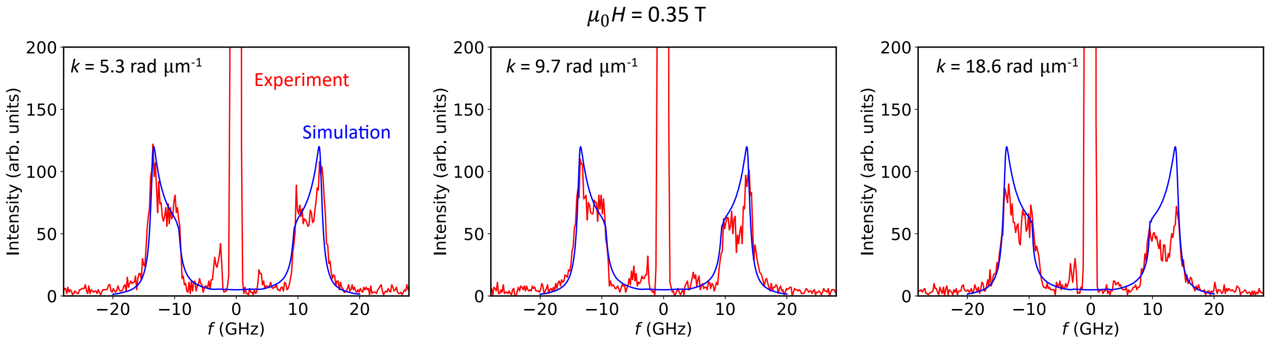

Supplementary Figure S13: Spin wave spectra at fixed k values for spin waves traveling along the stripes. The FFT intensity of the simulated spin wave propagation (in blue) and the BLS intensity (in red) are compared for three different k values: 5.3 rad μm^{-1}, 9.7 rad μm^{-1}, and 18.6 rad μm^{-1} for a magnetic field of 0.35 T applied orthogonal to the stripes (see Fig. 4a in the main text).

Propagation of spin waves orthogonal to stripes: We now simulate spin wave propagation orthogonal to the stripes (for experimental and simulation geometries, see Supplementary Fig. S14a and Supplementary Fig. S12b, respectively). In the BLS experiment, all the spin waves excited at every point within the gradient stripes illuminated by the $>$ 30 μm laser spot are measured. In contrast, in the simulation, spin waves are excited from a 10 nm linear source and propagate from that region (shown schematically in Supplementary Fig. S12b). Here, the spin waves do not propagate very far and those propagating in the +x and -x directions will experience opposite anisotropy gradients. In addition, the dispersion depends strongly on where the excitation region is placed. Despite these differences, we can still compare the simulated and measured spectra for this geometry. In Supplementary Fig. S14b, we observe that there is a single defined peak for both simulation and experiment. However, the simulated spin wave frequency is $\sim$1.5 GHz higher than the measured spin wave frequency. This offset can also be corrected for by including a uniaxial in-plane anisotropy in addition to the uniaxial out-of-plane anisotropy.

To justify the inclusion of an in-plane anisotropy, we performed longitudinal MOKE measurements of the gradient pattern shown in Supplementary Fig. S14a created in a similar CoFeB sample, which reveals strikingly different hysteresis loops when applying the field along the stripes and orthogonal to the stripes, seen in Supplementary Fig. S14c. The apparent asymmetry of the loops for positive and negative magnetic fields is due to the limited magnetic field range in the our MOKE magnetometer, which means we cannot fully saturate the film in-plane. This inability to saturate the film means that we cannot extract the value of the anisotropy field from these MOKE loops.

Nevertheless, the difference in shape of the hysteresis loops confirms there is an in-plane anisotropy in addition to the out-of-plane anisotropy.

By including a uniaxial in-plane anisotropy with the hard axis along the stripes (y direction in Supplementary Fig. S12b) with an anisotropy energy of $K_{\mathrm{IP}} = 4.3 \times 10^4$ J m^{-3}, the simulated frequency peak can be shifted to match that of the experimental data, as seen in Supplementary Fig. S14d for three different k values. We confirm that this additional anisotropy does not affect the spin wave dispersion for the geometry of spin waves propagating along a stripe (Supplementary Fig. S12a) by adding the same uniaxial term to the simulation for spin waves traveling along the stripe. In this case, there is no magnetization component along the in-plane hard axis because the magnetization is saturated along the y direction while the hard axis is along the x direction. As a result, this anisotropy does not affect the spin wave dispersion. This means that we can use the exact same anisotropy landscape to produce simulations of spin waves traveling along and orthogonal to the stripes that are comparable to the experimental data. The agreement between simulation and experiment provides additional evidence that DWLA is an effective tool to create designed magnetic landscapes.

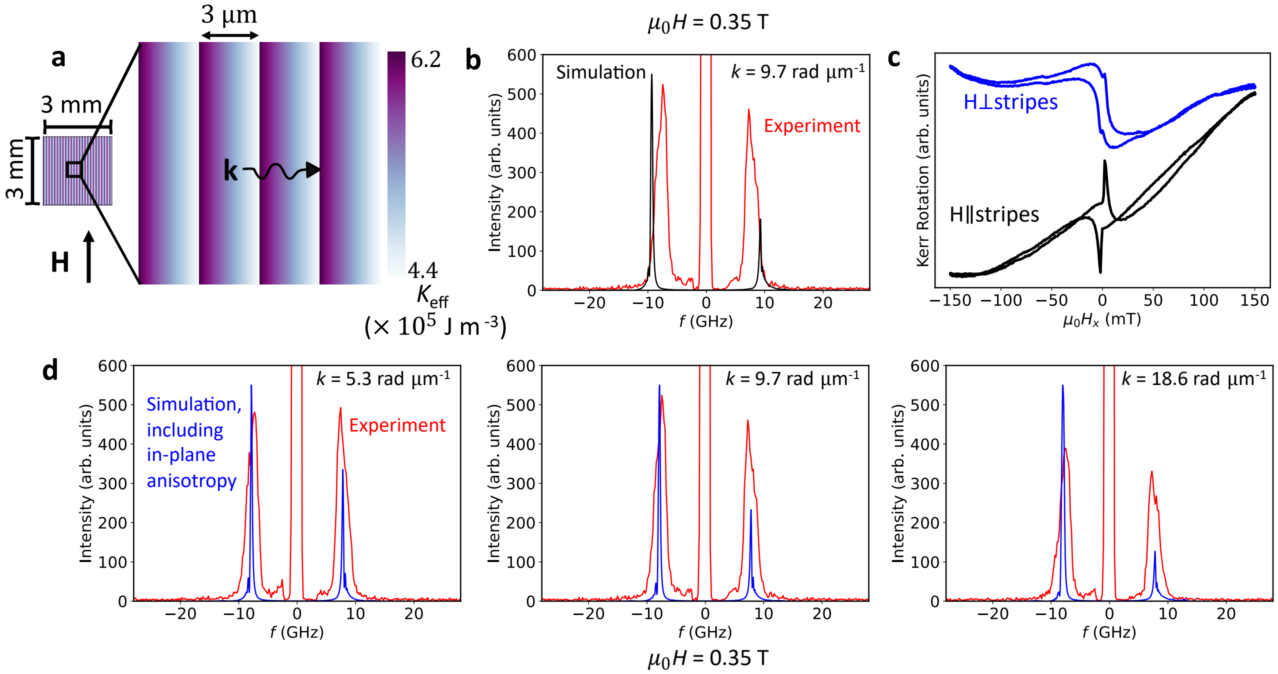

Supplementary Figure S14: Spin wave spectra at fixed k values for spin waves traveling orthogonal to the stripes. **a,** Schematic for the measurement geometry of spin waves that propagate orthogonal to the stripes (simulation geometry shown in Supplementary Fig. S12b), while the magnetic field is applied along the stripes. **b**, Comparison of spectra at 0.35 T and 9.7 rad μm^{-1} for simulation (in black) and the BLS intensity (in red). **c**, Longitudinal MOKE hysteresis loops of the gradient stripe pattern with the magnetic field applied along the stripes (in black) and orthogonal to the stripes (in blue). The significant difference in the shape of these hysteresis loops indicates an in-plane anisotropy in the patterned film. **d**, The FFT intensity of the simulated spin wave propagation including an in-plane anisotropy component (in blue) and the BLS intensity (in red) are compared for three different k values: 5.3 rad μm^{-1}, 9.7 rad μm^{-1}, and 18.6 rad μm^{-1} for an applied magnetic field of 0.35 T.

S4.3 Tunability of spin wave propagation bands

By changing the range of laser fluence in the gradient stripes, the gradient in anisotropy can be adjusted, and thus the frequency range of spin wave propagation can be modified.

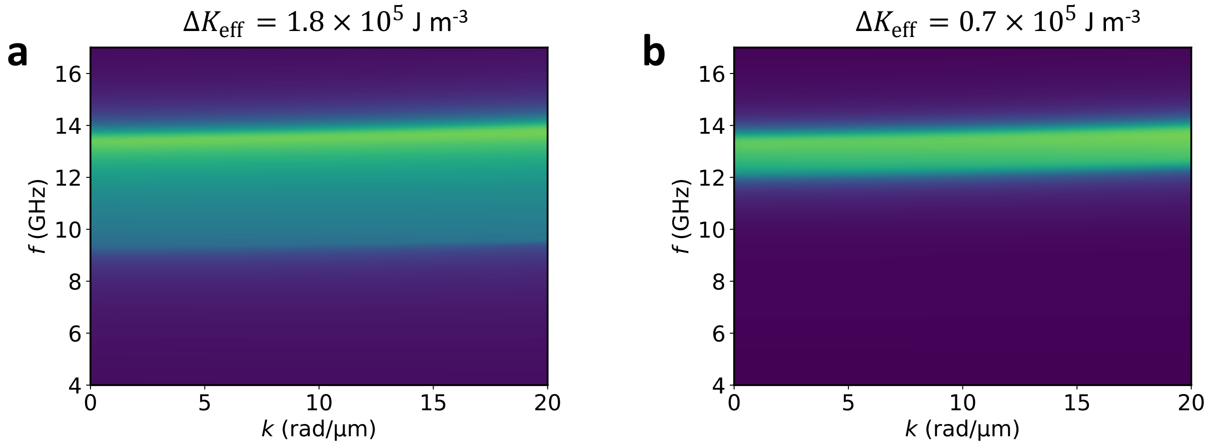

Supplementary Figure S15: Controlling the spin wave bandwidth by adjusting the anisotropy gradient of the stripes. a, Simulated dispersion of the magnetic landscape from the main text at an applied field of $\mu_0 H = 0.35$ T, with $K_{\rm eff} = 4.4 \times 10^5$ J m^{-3} $- 6.2 \times 10^5$ J m^{-3} across a 3 µm stripe. **b,** The same design with a weaker anisotropy gradient of $K_{\rm eff} = 4.4 \times 10^5$ J m^{-3} $-$ 5.1×10^5 J m^{-3} across a 3 µm stripe at $\mu_0 H = 0.35$ T, resulting in a smaller bandwidth of allowed spin wave frequencies.

S5 Unprocessed images of domain wall motion in CoFeB/Pt/Ru SAFs

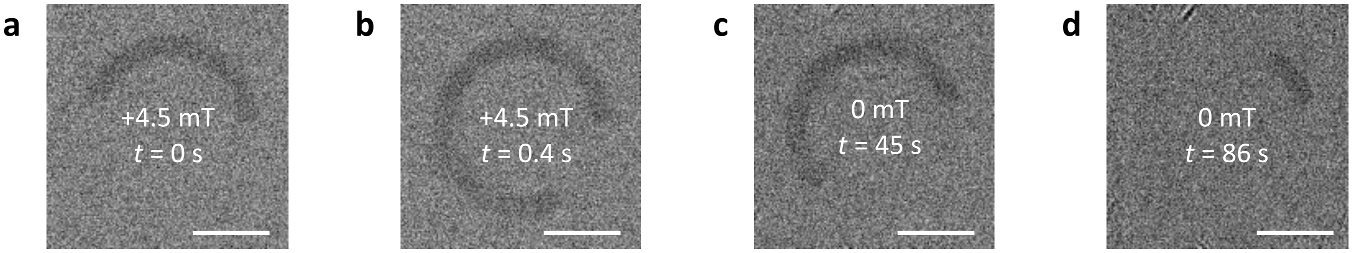

Supplementary Figure S16: Spontaneous domain wall motion along a curved anisotropy gradient. a-d, The original unprocessed Kerr microscope images used for those shown in Fig. 5b-e (scale bar = 10 µm). For the images in the main text, a Gaussian blur was applied and the contrast was enhanced.

S6 Further characterization of DWLA

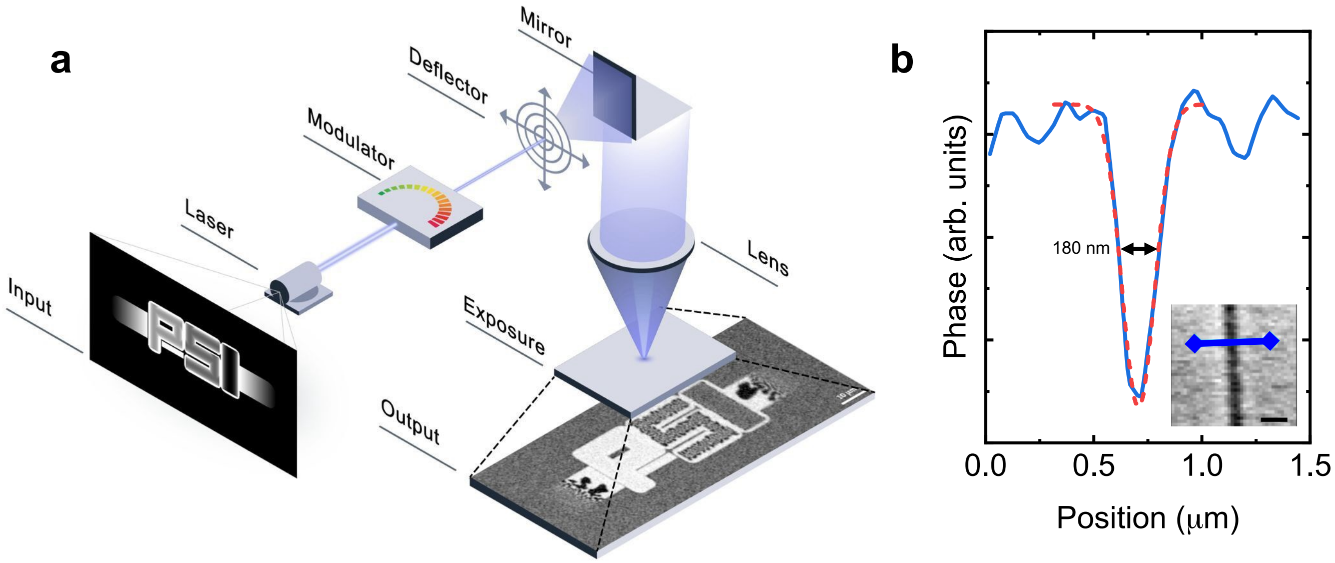

Supplementary Figure S17: Experimental details of DWLA. a, Procedure for local gradient laser annealing. The 2D varying grayscale intensity in the design is converted to variations in laser fluence by the modulator. The deflector rasters the laser on the film as the stage simultaneously moves in order to write a 2D pattern. **b,** We used DWLA (with a laser fluence of 6.5 J cm^{-2}) to create a ferromagnetically coupled area in the form of a line (visible as the dark region in the inset image) in a Co/Cr/Co SAF film. We then measured the area using magnetic force microscopy (MFM), and extracted the phase profile along the blue line shown in the inset. The resulting MFM contrast profile (solid blue line) and its Gaussian fit (dashed red line) reveal a full-width at half-maximum of approximately 180 nm, which provides an estimate of the minimum feature size. The nominal width of the exposed line was 50 nm in the design. Scale bar = 500 nm.

References

[1] Jaehun Cho, Jinyong Jung, Ka-Eon Kim, Sang-Il Kim, Seung-Young Park, Myung-Hwa Jung, and Chun-Yeol You. Effects of sputtering Ar gas pressure in the exchange stiffness constant of Co40Fe40B20 thin films. *Journal of Magnetism and Magnetic Materials*, 339:36, 2013.

[2] Jonas De Clercq, Jonathan Leliaert, and Bartel Van Waeyenberge. Modelling compensated antiferromagnetic interfaces with mumax3. *Journal of Physics D: Applied Physics*, 50(42):425002, 2017.
